# Intrastromal Corneal Ring Segments for Astigmatism Correction after Deep Anterior Lamellar Keratoplasty

**DOI:** 10.1155/2017/8689017

**Published:** 2017-08-29

**Authors:** Júlio C. D. Arantes, Sandro Coscarelli, Paulo Ferrara, Luana P. N. Araújo, Marcos Ávila, Leonardo Torquetti

**Affiliations:** ^1^Center of Reference in Ophthalmology, Federal University of Goiás, Goiânia, Brazil; ^2^Ennio Coscarelli Eye Clinic, Belo Horizonte, Brazil; ^3^Paulo Ferrara Eye Clinic, Belo Horizonte, Brazil; ^4^Altino Ventura Foundation, Recife, Brazil; ^5^Center of Excellence in Ophthalmology, Pará de Minas, Brazil

## Abstract

*Background*. To evaluate the change in corneal astigmatism after intrastromal corneal ring segment (ICRS) implantation in keratoconus patients with previous deep anterior lamellar keratoplasty (DALK). Design was a longitudinal, retrospective, interventional study. The study included 25 eyes of 24 patients with keratoconus who had DALK performed at least two years prior to ICRS implantation. All patients had a clear corneal graft with up to 8.00 D of corneal astigmatism and intolerance to contact lenses. The studied parameters were age, sex, corrected distance visual acuity (CDVA), maximum keratometry (K1), minimum keratometry (K2), spherical equivalent, and astigmatism. There was a statistically significant decrease in the postintervention analysis as follows: 3.5 D reduction in K1 (*p* < 0.001); 1.53 D in K2 (*p* = 0.005); and 2.52 D (*p* < 0.001) in the average *K*. The spherical equivalent reduced from −3.67 D (±2.74) to −0.71 D (±2.35) (*p* < 0.001). The topographic astigmatism reduced from 3.87 D preoperatively to 1.90 D postoperatively (*p* < 0.001). The CDVA improved from 0.33 (±0.10) to 0.20 (±0.09, *p* < 0.001). ICRS implantation is a useful option for the correction of astigmatism after DALK as it yields significant visual, topographic, and refractive results.

## 1. Introduction

Keratoconus is a noninflammatory, progressive ectatic disease characterized by thinning and protrusion of the cornea. It causes progressive myopia and irregular astigmatism, decreasing the quality of vision [1].

Treatment of keratoconus is indicated according to disease severity. Initially, correction is achieved by wearing glasses, followed by the fitting of rigid gas permeable contact lenses (RGPCL). When these methods fail, surgery is indicated to improve the corneal surface. [2]. When the cornea still appears transparent, the implantation of intrastromal corneal ring segments (ICRS) may be a successful option, and in many cases, it represents an alternative to corneal transplantation [3–6].

Penetrating keratoplasty (PKP) used to be the method of choice for treating ectatic corneal disease and has been the standard procedure for the keratoconus patient. A disadvantage of this method is its greater potential for graft rejection, which can lead to a significant reduction in visual acuity. [7–10]. The alternative approach has been deep anterior lamellar keratoplasty (DALK), in which a split-thickness graft is sutured to the receptor, sparing the host's endothelium and Descemet's membrane and avoiding the risk of endothelial rejection, with similar visual results [9]. DALK can be used in virtually all cases of corneal opacity not involving the endothelium, and it proved to be a valuable alternative to PKP for treating keratoconus [10–13].

The surgical duration and risk of ocular perforation have decreased, and the technique [11] has also provided an optical surface of excellent quality. In cases of a high residual post-DALK astigmatism, one alternative is intrastromal corneal ring segment (ICRS) implantation, which may be indicated for transparent corneas. [14, 15] The main goal is to reshape the cornea without removing tissue or weakening its central or paracentral region [16].

This study evaluated the use of intrastromal corneal ring segments implanted using a femtosecond laser as a surgical option for treating patients undergoing DALK due to keratoconus. No other studies were found that have evaluated the results of employing this implant in the same manner as proposed here.

## 2. Methodology

This was a retrospective, longitudinal study with secondary data collected from the medical records of patients diagnosed with keratoconus who underwent intrastromal corneal ring implantation for residual astigmatism correction after DALK.

An informed consent was given to all eligible patients prior to data collection, requesting permission for the research and use of data from their medical records relating to the pre- and postoperative periods. All bioethical principles were considered in accordance with the Declaration of Helsinki and Brazilian regulations.

The following were considered inclusion criteria: a clear and transparent corneal graft, a minimum of 2.50 diopters (D) and a maximum of 8.00 D of astigmatism, intolerance to contact lenses, and at least two years of follow-up after DALK before implanting the intrastromal corneal ring. Exclusion criteria were age under 18 years, having undergone DALK to treat a disorder other than keratoconus, any ocular surgery other than those proposed in the present study, history of corneal graft rejection, and any other associated ocular disease.

To evaluate the preoperative parameters, the data used were taken from the information recorded on the last visit prior to when surgery was indicated. All patients had completed at least one year of follow-up after ICRS implantation, and the second evaluation was based on information taken after this period had elapsed. Clinical variables were corrected distance visual acuity (CDVA), measured with the patient at a distance of 6 meters from the Snellen table, without cycloplegy. To analyze CDVA, the Snellen fractions were converted to LogMAR. Maximum keratometry (K1) and minimum keratometry (K2) were measured in diopters, using EyeSys topography (EyeSys Vision, Houston, United States). The spherical equivalent, in cylindrical diopters (DC), was obtained during refractometry performed with the patient at a distance of 6 meters from the Snellen table, without cycloplegy. Topographic astigmatism was measured in diopters, using EyeSys topography (EyeSys Vision, Houston, United States).

All ICRS implantations were performed by the same surgeon (SC). The procedures were performed from June 2012 to September 2013. These interventions were made using a standard technique, as previously describe [17].

DALK surgery was performed under peribulbar anesthesia. The cornea was partially trephined at a diameter of 7.5 mm. A radial incision was made with a diamond blade at the 90° meridian, which reached 90% of the corneal depth, from which stromal tunnels were created using a spatula. Air was injected into the stromal tunnels to create a deep cleavage between Descemet's membrane and the posterior stroma. Atraumatic Vannas scissors were used to remove the anterior stromal tissue along the edge of the existing partial trepanation. The donor cornea was trephined with a diameter 0.25 mm greater than that used in the recipient. The endothelium of the graft was completely removed. The donor button was fixed with 16 separate sutures using mononylon 10.0. Postoperatively, 0.3% moxifloxacin eye drops combined with 0.1% dexamethasone, four times a day for a period of six weeks, were prescribed. Lubricant eye drops were used several times a day, according to each patient's needs. The sutures removal began three months after surgery.

**Figure 1:**
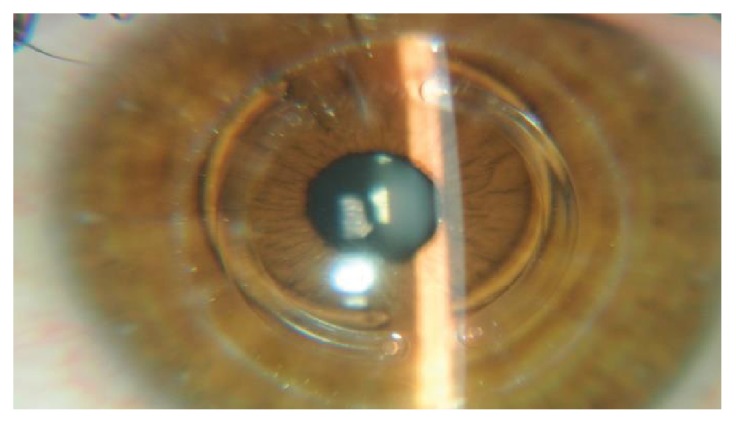
Transparent cornea one week after the ICRS implant.

The ICRS surgical implantation procedure was performed under topical anesthesia. The Purkinje reflex was chosen and marked with the tip of a Sinskey hook. A 5 mm corneal marker was used to locate the exact area of the channel for the ICRS implant. The depth of the tunnel was set at 75% of corneal thickness at its thinnest area. An incision was made on the steepest topographic axis, with one or two segments implanted according to the distribution of the ectatic area on the corneal surface and the degree of topographic astigmatism. A femtosecond laser frequency of 60 kHz (LDV Z6, Ziemer, Switzerland) was used to create the ring segment tunnels. Special attention was given to centralizing the suction ring to mark the center point to minimize decentration. The inner diameter of the channel was set at 4.4 mm, and the outer diameter was 5.6 mm; the energy used to create the channel was 1.30 J. The time taken to create the channel with the femtosecond laser was seconds. The ICRS were implanted immediately after creation of the channel (before the disappearance of the bubbles) and were inserted using a modified McPherson forceps. The segments were correctly positioned with the aid of a Sinskey hook. Postoperative procedures included the application of 0.3% moxifloxacin eye drops combined with 0.1% dexamethasone, four times daily for two weeks. Furthermore, patients received topical lubricants to be applied four times a day for at least three months (Figure 1).

All patients were evaluated within the service's routine, which offered return visits on the first and seventh postoperative day, after one and six months, and annually thereafter; for analysis purposes, the postoperative data were considered after one year of follow-up.

Data were analyzed using the Statistical Package for the Social Sciences (SPSS), version 19.0. The Kolmogorov-Smirnov test was applied to identify the distribution of continuous variables, and Student's *t*-test, the Welch transformation, and the nonparametric Mann–Whitney tests were also applied. All tests considered a significance level of 5% and a 95% confidence interval.

## 3. Results

This study included 25 eyes of 24 patients, 14 patients were male (58.33%) and 10 were female (41.66%), aged 20–54 years (mean: 35.96; standard deviation: ±8.69 years). The indication for ring implantation was to correct residual astigmatism after DALK. Twenty-three patients had one eye treated, and only one patient had both eyes treated.

**Figure 2:**
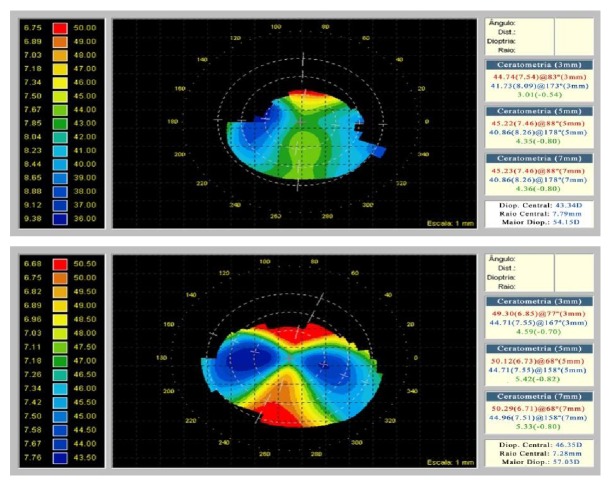
Preoperative and postoperative corneal topography after ICRS implantation.

All clinical variables showed a statistically significant decrease from preoperative to postoperative. There was a reduction of 3.50 D in K1 (*p* < 0.001) and 1.53 D in K2 (*p* = 0,005), and the reduction in the mean *K* was 2. D (*p* < 0.001) (Figure 2).

The spherical equivalent (SE) reduced from −3.67 D (±2.74) before the procedure to −0.71 D (±2.35) after the procedure (*p* < 0.001). Topographic astigmatism reduced from 3.87 D preoperatively to 1.90 DC postoperatively (*p* < 0.001) (Table 1). The corrected distance visual acuity (CDVA), in LogMAR, increased from 0.33 (±0.10) to 0.20 (±0.09; *p* < 0.001).

**Figure 3:**
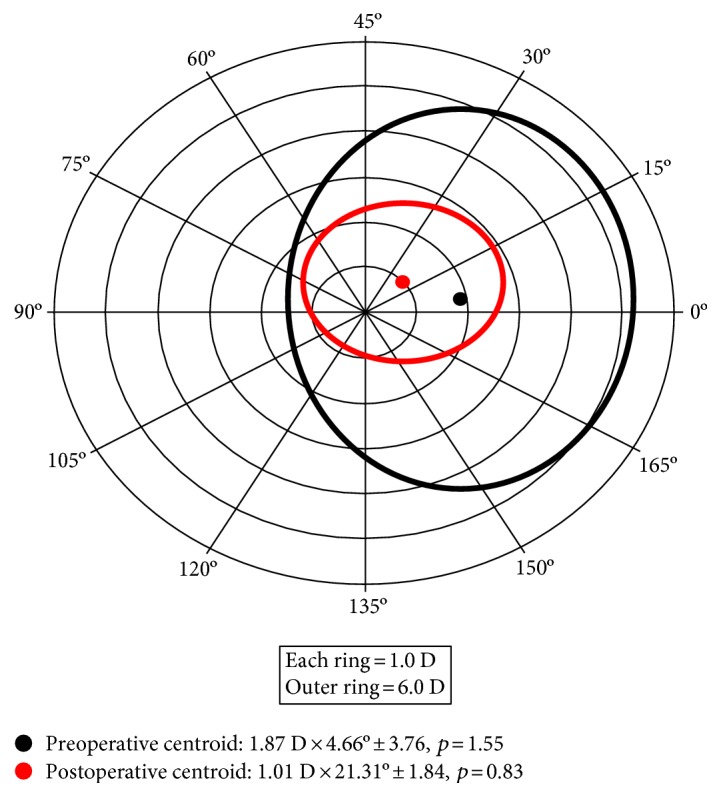
Double-angle *plot* of preoperative and postoperative refractive astigmatism. The standard deviation is represented by the area around the centroid (*p*=shape factor). Each ring = 1.0 D/outer ring = 6.0 D.

Vector analysis (double-angle plot) revealed a significant decrease in the centroid between the preoperative and postoperative data in both topographic astigmatism and refractive astigmatism (Figure 3).

The preoperative refractive astigmatism centroid was 1.87 D × 4.66° ± 3.76 (*p* = 1.55), and the postoperative centroid was 1.01 D × 21.31° ± 1.84 (*p* = 0.83). After implantation of the ICRS, the refractive astigmatism centroid was 0.86 D, and the standard deviation in astigmatism was reduced by a factor of 1.92 (3.76 D/1.84 D). Relocation of the centroid nearer to the origin and contraction of the ellipse in the double-angle plots showed improvement (Figure 3).

**Figure 4:**
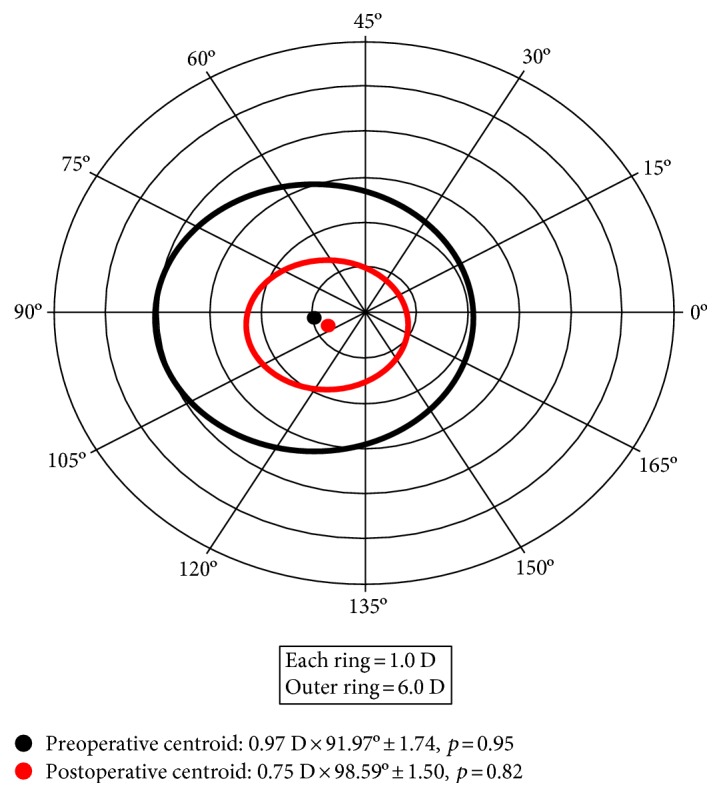
Relationship between preoperative and postoperative keratometric astigmatism. The standard deviation is represented by the area around the centroid (*p*=shape *factor*). Each ring = 1.0 D/outer ring = 6.0 D.

Figure 4 shows the double-angle plot for preoperative and postoperative keratometric astigmatism. The preoperative centroid was 0.97 D × 91.97° ± 1.74, (*p* = 0.95), and the postoperative centroid was 0.75 D × 98.59° ± 1.50, (*p* = 0.82). Although there was a reduction in mean keratometric astigmatism, it was considerably smaller than the decrease in refractive astigmatism.

## 4. Discussion

The most common cause of decreased vision after corneal transplantation is the astigmatism. It is commonly accepted that the average postoperative cylinder after keratoplasty varies from three to five diopters; [18] about 10–27% of patients undergoing corneal transplantation evolve with high astigmatism, and for high astigmatism, it is understood as the refractive cylinder of more than four diopters (D) [19].

**Figure 5:**
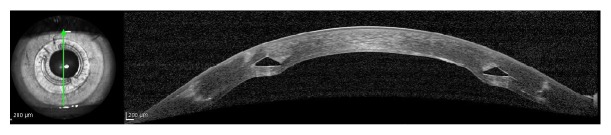
Optical coherence tomography (OCT) of the cornea one year after the procedure, showing the position of the ICRS.

In the present study, using ICRS implantation to treat high astigmatism after the DALK significantly reduced *K* values, spherical equivalent, and the topographic astigmatism. Tunnels made with the femtosecond laser have an advantage over those implanted using the manual technique because they cause lower traction at the junction where the recipient cornea meets the donor button, avoiding possible dehiscence in the surgical wound [3] (Figure 5()).

The choice of a Ferrara ICRS with a 5 mm optical zone also has advantages over choosing those that are implanted in a 6 or 7 mm optic zone, as the former theoretically causes greater corneal flattening because the refractive results are inversely proportional to the implant diameter [16]. Furthermore, a smaller optical zone provides greater distance between the ICRS and graft/host junction, reducing the chance of stromal neovascularization/dehiscence of the junction. In turn, a smaller optical zone increases the risk of postoperative halos. Excimer laser photorefractive keratectomy (PRK) and laser in situ keratomileusis (LASIK) can also be used to treat posttransplant astigmatism [20].

The fact that the ICRS implant did not affect the central region of the cornea, avoiding the risk of opacity on the visual axis and the reversibility of this method, means that this can be considered theoretically more beneficial than the correction of astigmatism using LASIK, which has limited efficacy due to the stromal thickness of the graft and the high remaining ametropia [20].It also does not provide results as good as those seen in corneas that have never been treated, with less predictability and greater chance of complications such as epithelialization defects, flap displacement, dry eye, and corneal graft failure [18, 21, 22]. PRK can cause a significant haze in corneal grafts and induce progressive astigmatism [20].

Another surgical option for the treatment of post-DALK astigmatism is the implantation of a phakic intraocular lens (PIOL). Barraquer and Rodriguez-Barraquer [23] evaluated the Artisan PIOL implant in the treatment of high myopia after PKP. In that study, there was an improvement in CDVA without significant loss of endothelial cells in the first six postoperative months [23].

Another study demonstrated that a PIOL implant in post-PKP eyes reduces refractive error and improves the CDVA, in addition to being a predictable and stable method. However, even if the refractive error is significantly reduced, corneal abnormalities will still be present. Alternatively, an ICRS implant decreases these abnormalities, improving the quality of vision. In addition, as it is a less invasive corneal procedure, the risks related to intraocular surgery are avoided [24]. Alfonso et al. [25] evaluated the efficacy and safety of the implantable collamer posterior chamber intraocular lenses to correct refractive errors that occurred after PKP in 15 eyes of 15 patients. In this study, no eye lost more than one line of vision, two eyes gained one line, five eyes gained more than one line, and eight eyes remained with unchanged vision [25, 26].

In the current study, when evaluating the CDVA 12 months after ICRS implantation, stability was observed in 28% (seven eyes); 36% (nine eyes) gained one line of vision; 20% (five eyes) gained two lines of vision; 8% (two eyes) gained three lines of vision; and 8% (two eyes) gained four lines of vision. No eyes lost lines of vision.

Other scientific studies using a post-PKP manual intrastromal corneal ring implant technique showed *K* reduction values higher than those found in our study. A study conducted in Spain, with the participation of nine patients (nine eyes), found a reduction in K1 values greater than 5 D and a reduction in K2 greater than 2 D, while the mean *K* decreased by more than 4 D [3]. In another publication using a similar technique conducted in Brazil evaluating patients (59 eyes), *K* values were also reduced by more than the amount found in our study, with the maximum *K* decreasing by 3.92 D and the minimum *K* by 2.44 D [17].

In another study with 30 patients (32 eyes) using a femtosecond laser for post-PKP intrastromal corneal ring implantation, lower reductions in K2 (0.53 D) and K1 (3.65 D) were found, a result similar to that of our study [14].

Arriola-Villalobos [27] found that the spherical equivalent decreased by 3.05 D, and Coscarelli et al. [16] reported a decrease of 3.68 D. These values are similar to those found in our study, in which we observed a decrease of 2.96 D. In the study of Lisa et al. [13], a smaller reduction in the spherical equivalent of 1.67 D was observed.

Topographic astigmatism decreased 1.97 in our study, whereas in another similar study, a decrease of 2.59 was obtained [9].

We hypothesize that the results, in terms of reduction of keratometry and astigmatism, are less significant in cases of ICRS implantation after DALK, in comparison of ICRS after PKP due to biomechanics properties of the cornea The preservation of Descemet's membrane and endothelium in DALK provides an additional strength to the graft, comparing to PKP, which in turns, leads to less response to corneal flattening/remodeling after ICRS implantation in these cases. Moreover, the irregularity at host-donor interface may play a role in the corneal changes after ICRS implantation after PKP.

In our investigation, there were no complications during the surgery or postoperative follow-up. The use of the femtosecond laser in the construction of stromal tunnels is safer than the manual technique, providing a significant reduction in complications such as ICRS extrusion because of the accuracy in implant depth [27–29].

One limitation of the study was the lack of standardization in the medical records, making the data difficult to collect and increasing the time required to populate the database, although all the information necessary for the proposed evaluations were obtained in the documents.

One of the strengths of this study is the fact that no other studies were found that evaluates the results of ICRS implantation using a femtosecond laser in patients previously undergoing DALK due to keratoconus. The number of patients selected was lower than ideal due to the rigorous selection of the studied eyes. The strict inclusion and exclusion criteria limited the sample to 25 eyes, but statistical significance was achieved.

The results of this study indicate that ICRS implanted using a femtosecond laser is a surgical treatment option for patients having undergone DALK due to keratoconus that offers significant visual, topographic, and refractive results. Prospective studies with longer follow-up periods are needed to verify the long-term safety and stability of the procedure.

### 4.1. What Was Known before This Study

The use of a femtosecond laser for intrastromal corneal ring implantation is a safe option for treating astigmatism after PKP.

### 4.2. What This Study Has Added

This is the first published study of ICRS implantation with a femtosecond laser to correct high astigmatism in eyes that were previously subjected to DALK due to keratoconus.

In a series of 25 eyes, the ICRS implant produced a significant reduction in topographic astigmatism and improved the CDVA in patients previously undergoing deep anterior lamellar keratoplasty.

## 5. Synopsis

ICRS implantation can successfully improve the visual acuity and reduce the astigmatism after deep anterior lamellar keratoplasty.

## Supplementary Material

The attached table shows the date of surgery, the operated eye, the preoperative and postoperative refraction, the preoperative and postoperative visual acuity, and the characteristics of the corneal ring segments that were implanted in each patient.

## Figures and Tables

**Table 1:** Preoperative and postoperative data after ICRS implantation.

	Preop		Postop		
					*p* ^∗^
	Mean	SD	Mean	SD	
Maximum keratometry	47.20	±2.50	43.70	±3.45	**<0.001**
Minimum keratometry	43.33	±2.00	41.80	±3.53	0.005
Mean keratometry	45.26	±2.03	42.74	±3.44	**<0.001**
Spherical equivalent	−3.67	±2.74	−0.71	±2.35	**<0.001**
Topographic astigmatism	3.87	±1.95	1.90	±1.16	**<0.001**

^∗^Student's *t*-test for paired samples, *p* value. SD: standard deviation.
