# Supplementary material for: Intrastromal Corneal Ring Segments for Astigmatism Correction after Deep Anterior Lamellar Keratoplasty
Source: J Ophthalmol. 2017 Aug 29;2017:8689017. doi: 10.1155/2017/8689017 (PMC5603116; doi:10.1155/2017/8689017)
Supplement: Supplementary file 1 — The attached table shows the date of surgery, the operated eye, the preoperative and postoperative refraction, the preoperative and postoperative visual acuity, and the characteristics of the corneal ring segments that were implanted in each patient. [file 8689017.f1.pdf]

PACIENTE	DATA CIRURGIA	OLHO	REFRAÇÃO PRÉ	REFRAÇÃO PÓS	AV PRÉ	AV PÓS	SEGMENTO
SAULO SAMUEL DE PAIVA VILELA	20/06/12	OE	-1.25 (-4.75X150º)	+1.00 (-1.50X180º)	20/40	20/30	2X160/20
WILLIAN MACHADO OLIVEIRA	20/06/12	OE	-6.50 (-3.50X140º)	-2.50 (-2.00X15º)	20/40	20/30	2X160/20
ANDREIA MARIA MONTEIRO SILVA	04/07/12	OD	-1.00 (-7.00X25º)	+2.50 (-3.50X20º)	20/50	20/30	2X160/20
CARLOS GILBERTO ARCANGELO	04/07/12	OD	-2.00 (-8.00X130º)	+1.00 (-4.00X155º)	20/60	20/40	2X160/20
ROSE DA CONCEIÇÃO DOS ANJOS	08/08/2012	OD	PLANO (-6.00X60º)	+0.50 (-4.00X40º)	20/30	20/30	1X160/20
GUSTAVO GUIMARAES FONSECA	08/08/2012	OD	PLANO (-8.00X50º)	+3.00 (-4.00X45º)	20/40	20/40	2X160/20
ODIM DONATO FILHO	17/08/2012	OD	+1.00 (-7.00X90°)	+2.50 (-4.00X93)	20/40	20/30	2X160/20
PRISCILA GONÇALVES GARCIA	22/08/2012	OD	-3.50 (-1.50X45º)	-1.50 (-2.00X15º)	20/30	20/30	1X160/20
JOAO BOSCO NASCIMENTO SOUZA SANTOS	05/09/2012	OE	+2.00 (-5.00X120º)	+1.50 (-1.50X15º)	20/50	20/20	2X140/20
JULIA MARIA BARBOSA	05/09/2012	OE	-2.50 (-3.00X25º)	+2.00 (-2.00X90º)	20/30	20/30	2X160/20
ELIZA MARIA SEVERINO FIGUEIREDO	26/09/2012	OE	-2.00 (-3.00X80º)	+0.50 (-2.00X75º)	20/40	20/30	2X160/20
MATHEUS LEONIDAS SILVA	03/10/2012	OD	PLANO (-3.50X145º)	+1.00 (-2.00X30º)	20/30	20/25	2X160/20
JORGE DA PENHA DE FREITAS	11/11/2012	OD	PLANO (-5.50X15º)	+2,50 (-2.00X15º)	20/40	20/30	2X160/20
ELAINE APARECIDA RESENDE DIAS	24/10/2012	OE	+2.50 (-5.00X145º)	+4.00 (-0.50X105º)	20/40	20/40	2X160/20
ANGELICA DAS GRACAS GOMES SANTOS	24/10/2012	OE	+2.00 (-8.00X150º)	+3.50 (-3.50X155º)	20/50	20/50	2X160/20
SUELI ELISABETE DIAS	24/10/2012	OE	+0.75 (-6.00X10º)	+1.75 (-1.75X50º)	20/50	20/40	2X160/20
SIRLEI JULIANA DIAS	24/10/2012	OE	-4.25 (-3.75X150º)	-2.00 (-1.50X135º)	20/40	20/30	2X160/20
JASON FRANCIS RODRIGUES DA SILVA	31/10/2012	OE	-2.50 (-6.00X180º)	-1.00 (-3.50X15º)	20/40	20/25	2X160/20
JASON FRANCIS RODRIGUES DA SILVA	14/11/2012	OD	+2.00 (-6.00X180º)	+2.25 (-1.75X180º)	20/50	20/20	2X140/20
MARCUS VINICIUS REIS DE FREITAS	14/10/2012	OE	-2.75 (-3.00X20º)	-0.75 (-2.00X10º)	20/30	20/30	1X160/20
SALIME GUALBERTO DA ROCHA JUNIOR	10/09/2013	OE	-6.00 **(**-6.00X175°**)**	-4.25 **(**-2.00X65°**)**	20/40	20/25	2X160/20
LARA DRUMMOND PAIVA	11/07/2013	OE	+2.00 **(**-6.00X20°**)**	+2.50 **(**-2.00X20°**)**	20/40	20/30	2X140/20
TIAGO SOUSA RAMOS	11/05/2013	OE	+4.50 **(**-4.50X20°**)**	-0.50 **(**-3.25X55°**)**	20/50	20/30	2X140/20
JAMERSON DE MATOS ASSIS SOUZA	11/03/2013	OE	-5.00 **(**-8.00X30°**)**	-3.50 **(**-5.00X180°**)**	20/80	20/40	2X160/20
ANTONIO FERREIRA DOS SANTOS FILHO	09/01/2013	OE	-3.00 **(**-4.50X20°**)**	-2.00 **(**-2.00X120°**)**	20/60	20/30	2X160/20

CERATOMETRIA PRÉ	CERATOMETRIA PÓS	IDADE
48.60@62º / 46.66@152º	47.71@131º / 47.22@41º	39
44.96@56º / 43.45@146º	41.49@88º / 40.99@178º	29
48.94@116º / 44.91@26º	44.95@111º / 42.34@21º	31
49.43@33º / 45.67@123º	48.30@67º / 47.15@157º	50
45.74@155º / 40.81@65º	43.49@133º / 40.12@43º	44
45.86@139º / 41.46@49º	39.69@133º / 35.73@43º	35
47.57@179º / 41.64@89º	39.24@10º / 36.26@100º	50
47.82@133º / 45.84@43º	46.47@157º / 45.18@67º	27
42.73@31º / 40.37@121º	40.65@80º / 39.58@170º	31
46.08@105º / 44.99@15º	42.50@180º / 40.50@90	36
45.28@169º / 42.88@79º	43.27@83º / 42.78@173º	48
43.88@53º / 42.19@143º	40.21@116º / 39.70@26º	24
44.07 @ 23° / 47.87 @ 113°	41.94@175° / 43.32@85°	54
44.57@48º / 42.50@138º	38.69@11º / 38.15@101º	32
47.34@59º / 41.66@149º	39.82@63º / 37.15@153º	33
45.99@97º / 41.90@7º	43.26@93º / 41.02@3º	45
46.71@60º / 43.80@150º	42.36@104º / 41.97@14º	35
51.93@69º / 45.23@159º	47.37@115º / 46.30@25º	20
45.93@106º / 40.95@18º	40.05@98º / 38.13@8º	20
48.99@110º / 46.78@20º	44.54@67º / 42.44@157º	36
44.21@@168° / 49.63@78°	44.99@174 / 49.18@84°	34
42.30@10° / 48.60@100°	44.90@15° / 47.10@105°	35
41.64@39° / 43.49@27°	44.04@39° / 46.16@129°	36
41.39@23° / 49.12@113°	38.19@180° / 42.11@90°	37
46.0@110° / 52.9@20°	48.0@120° / 50.9@10°	38
